# Conflicting responses with simultaneous atrioventricular pacing. What is the mechanism?

**DOI:** 10.1016/j.ipej.2024.08.004

**Published:** 2024-08-22

**Authors:** Suresh Kumar Sukumaran, Anish Bhargav, Sridhar Balaguru, Raja J. Selvaraj

**Affiliations:** Department of Cardiology, JIPMER, Pondicherry, India

## Case

A 60-year-old woman presented with recurrent episodes of palpitations, documented short RP, narrow QRS tachycardia and absence of preexcitation in the electrocardiogram during sinus rhythm. During an electrophysiology study, programmed stimulation induced a narrow QRS tachycardia with cycle length of 380 ms, VA interval of 164 ms and earliest atrial activation in the His region. Ventricular overdrive pacing failed to entrain the atrium even with isoprenaline infusion and atrial burst pacing repeatedly terminated the tachycardia. Difference in AH interval with pacing and SVT was 27 msec. Simultaneous atrial and ventricular pacing was done with atrial pacing from the high right atrium and showed a His signal as the first return electrogram suggestive of atrioventricular nodal reentrant tachycardia (AVNRT). The manoeuvre was repeated with atrial pacing from the proximal coronary sinus and showed an atrial signal as the first return electrogram suggestive of atrial tachycardia (AT). What is the explanation for the conflicting results of the two pacing maneuvers?

## Discussion

**Fig. 1:**
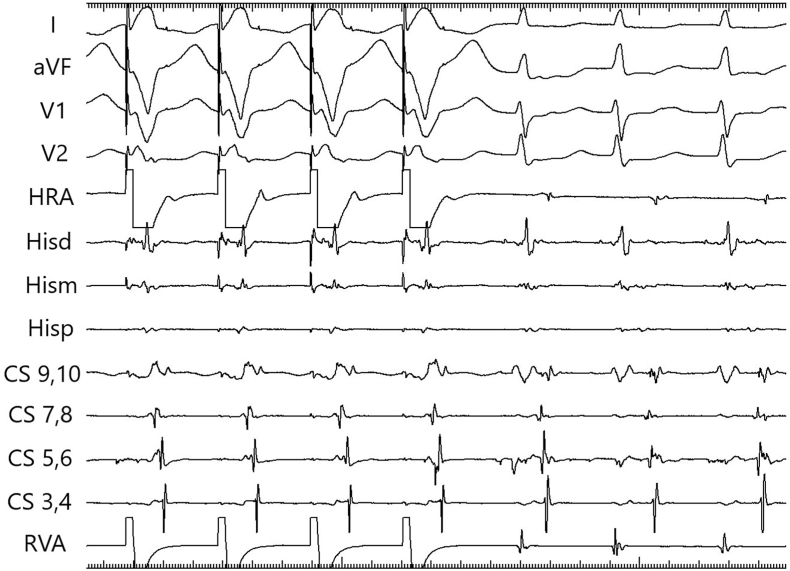
Simultaneous atrial and ventricular pacing at a cycle length of 310 msec. The first return signal is His electrogram in the His channel.

**Fig. 2:**
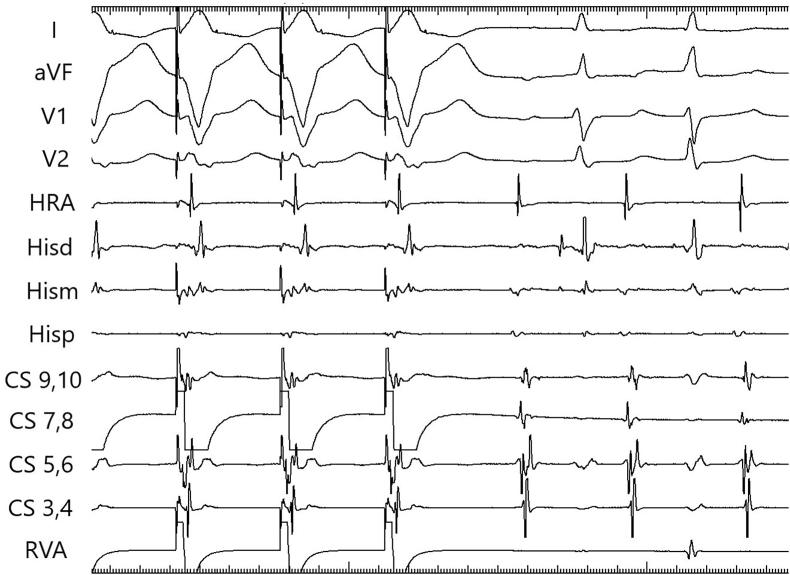
Simultaneous atrial and ventricular pacing at a cycle length of 350 msec. The first return signal is A in the His channel.

The short RP tachycardia with central atrial activation and a VA interval of 164 msec can be atypical AVNRT, AT or atrioventricular reentrant tachycardia (AVRT) [1]. Inability to entrain the atrium with ventricular overdrive pacing rules out AVRT. When ventricular overdrive pacing fails there are only a limited number of manoeuvres to differentiate the tachycardia mechanism. The difference in AH interval with pacing and SVT was 27 msec and was unclassifiable [2]. Atrial pacing was attempted to assess for linking, but always terminated the tachycardia. Simultaneous atrial and ventricular pacing can separate atrial tachycardia from atypical AVNRT. In a study of 80 patients, the first return intracardiac signal after simultaneous atrial and ventricular pacing was always A in atrial tachycardia and His in AVNRT [3]. (See Fig. 1, Fig. 2)

There were small changes in tachycardia cycle length accounting for the different cycle lengths during the two manoeuvres. However, there was no abrupt change in cycle length and atrial activation remained the same, confirming that the mechanism of the tachycardia remained the same throughout.

**Fig. 3:**
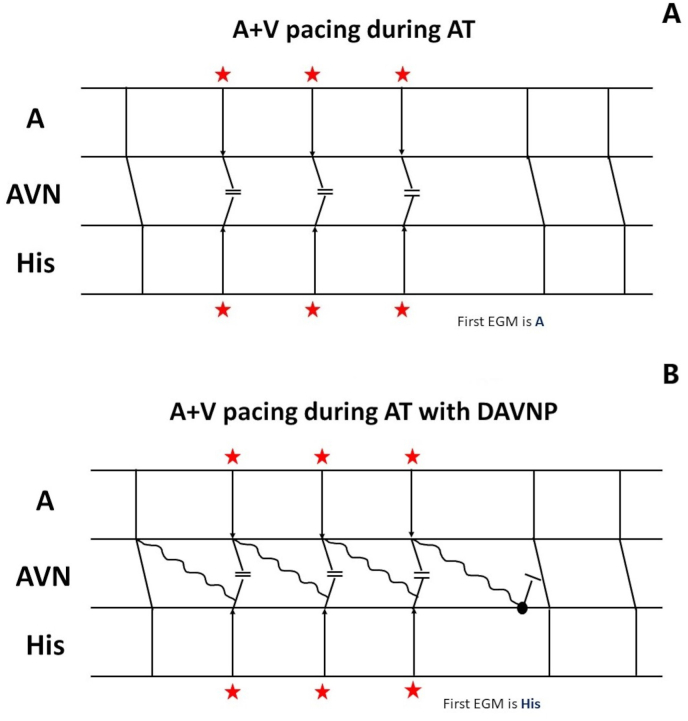
Fig. 3A Ladder showing simultaneous atrial and ventricular pacing during atrial tachycardia. The first return signal is A Fig. 3B Simultaneous atrial and ventricular pacing during atrial tachycardia with dual AV nodal physiology. With a dual AV nodal physiology, the last paced atrial stimulus can conduct antegrade through the slow pathway and was able to depolarize the His before the atrial focus. Hence the first return signal is His.

In atrial tachycardia, the last atrial paced impulse is unable to conduct to the ventricle because of simultaneous ventricular pacing and therefore tachycardia resumes with an A (Fig. 3, panel A). The only plausible explanation for His to be seen as the first return signal in atrial tachycardia is the presence of a dual AV nodal physiology. With a dual AV nodal physiology, the last paced atrial stimulus can conduct antegrade through the slow pathway (Fig. 3, panel B). The emergence of His or A as the first intracardiac signal after cessation of pacing depends on the ability of this slow pathway conduction to depolarize the His before the recovery of the atrial focus.

In the first instance, we presume that His was seen as the first intracardiac signal because of slow pathway conduction and the atrial focus was probably overdrive suppressed by the faster atrial pacing. In the second instance, pacing at a longer cycle length allowed the atrial focus to depolarize before the slow pathway could depolarize the His. Due to the inability to confirm a diagnosis, slow pathway ablation was first attempted, but failed to eliminate tachycardia. Subsequently the earliest atrial activation was mapped to the anteroseptal region and ablation within the non-coronary aortic sinus was successful in making tachycardia non-inducible.

In the situation where ventricular overdrive pacing is unable to entrain the atrium, simultaneous atrial and ventricular pacing is a valuable manoeuvre to differentiate atrial tachycardia from atrioventricular nodal reentrant tachycardia. However, in the presence of dual AV nodal physiology, this manoeuvre may not be reliable to distinguish the two mechanisms.

## Ethical statement

I confirm that the patient is 60 years old (>18 years) and has given written informed consent for the procedure. The patient's identity is anonymous in this manuscript. I confirm my ethical/consent statement for this publication.

## Declaration of competing interest

The authors declare that they have no known competing financial interests or personal relationships that could have appeared to influence the work reported in this paper.
